# Cardiovascular Manifestations of Patients with Long COVID

**DOI:** 10.3390/diagnostics15141771

**Published:** 2025-07-13

**Authors:** Gordana Krljanac, Milika Asanin, Mihajlo Viduljevic, Stefan Stankovic, Kristina Simatovic, Ratko Lasica, Olga Nedeljkovic-Arsenovic, Ruzica Maksimovic, Slavisa Zagorac, Ana Savic-Radojevic, Tatjana Djukic, Goran Stevanovic, Vedrana Pavlovic, Tatjana Simic

**Affiliations:** 1 Clinic of Cardiology, University Clinical Centre of Serbia, 11000 Belgrade, Serbia; 2 Faculty of Medicine, University of Belgrade, 11000 Belgrade, Serbia; 3 Centre for Radiology and Magnetic Resonance Imaging, University Clinical Centre of Serbia, 11000 Belgrade, Serbia; 4 Clinic of Orthopedic Surgery, University Clinical Centre of Serbia, 11000 Belgrade, Serbia; 5 Institute of Medical and Clinical Biochemistry, 11000 Belgrade, Serbia; 6 Clinic of Infectious and Tropical Diseases, Clinical Centre of Serbia, 11000 Belgrade, Serbia; 7 Institute for Medical Statistics and Informatics, 11000 Belgrade, Serbia; 8 Department of Medical Sciences, Serbian Academy of Sciences and Arts, 11000 Belgrade, Serbia

**Keywords:** long COVID, COVID-19, cardiovascular diseases, multimodality imaging, echocardiography

## Abstract

**Background**: This study investigates the potential mechanisms behind changes in cardiac structure and function in long COVID patients. **Methods**: This study involved 176 consecutive outpatients in follow-up care (average age 55.9 years; 58.5% male) who experienced symptoms for over 12 weeks (average 6.2 ± 2.7 months), following coronavirus infection (COVID-19). **Results**: The patients with long COVID and cardiovascular manifestations were significantly more hospitalized (88.5% vs. 75.9%) and had longer hospital stays. Significant echocardiography changes were observed in the left ventricular ejection fraction (LVEF) (59.6 ± 5.4% vs. 62.5 ± 3.8%); longitudinal strain (LS) in the sub-endocardium and intra-myocardium layers (−20.9 vs. −22.0% and −18.6 vs. −19.5%); circumferential strain (CS) in the sub-epicardium layers (−9.6 vs. −10.5%); and CS post-systolic shortening (CS PSS) (0.138 vs. 0.088 s). Additionally, pathological cardiac magnetic resonance (CMR) findings were seen in 58.2% of the group of patients with long COVID and cardiovascular manifestation; 43.3% exhibited positive late gadolinium enhancement (LGE), 21.0% had elevated native T1 mapping, and 22.4% had elevated native T2 mapping. **Conclusions**: Most patients with long COVID showed structural and functional changes in their cardiovascular systems, primarily caused by prolonged inflammation. Using multimodality imaging is important for uncovering the mechanisms to predict chronic myocarditis, early-stage heart failure, and pre-ischemic states, which can lead to serious complications. Recognizing the specific cardiovascular phenotypes associated with long COVID is essential in order to provide timely and appropriate treatment.

## 1. Introduction

Millions of coronavirus infection (COVID-19) patients have survived the infection; however, studies estimate that one in eight individuals with COVID-19 experiences persistent somatic symptoms attributable to a previous COVID-19 infection, commonly referred to as long COVID [1,2]. Long COVID is defined as the continuation of new or ongoing symptoms for four weeks or more after the onset of acute COVID-19 infection, which cannot be attributed to any other illness [3]. It can also be characterized by symptoms persisting for at least 12 weeks in a relapsing and remitting or progressive manner, affecting one or more organ systems [4].

Long COVID is a complex condition characterized by a variety of symptoms affecting multiple organ systems [5]. The symptoms associated with cardiovascular manifestation can vary widely due to differences in prevalence estimates, which depend on factors such as the source of enrollment, age and sex, vaccination rates, the presence of severe acute respiratory syndrome coronavirus-2 (SARS-CoV-2) variants, co-existing health conditions, and sociodemographic factors [6]. The most common cardiovascular symptoms include fatigue, shortness of breath, effort intolerance, chest pain, syncope, and palpitations.

The symptoms can be a result of various pathophysiological overlapping mechanisms, such as direct viral toxicity, autoimmune responses, inflammation of the heart muscle and pericardium, and ischemic diseases caused by the thrombosis of blood vessels and endothelial dysfunction [6,7]. The direct tissue damage induced by chronic inflammation can be induced by the persistence of the virus or viral residuum in tissues, the development or worsening of autoimmunity, the enhanced production or release of proinflammatory cytokines and chemokines, microbial dysbiosis, and the reactivation of latent viral infections unrelated to COVID-19 [8]. These mechanisms may drive one or more pathophysiological models. Endothelial dysfunction is now recognized as a central factor in long COVID. The development of inflammation activates endothelial cells, which may cause an intense inflammatory response resulting in the production of reactive oxidative species and the disruption of the balance between procoagulant and fibrinolytic factors in the vascular system [9]. Pathological autopsy studies suggest a pattern of cardiac dysfunction and myocardial injury following COVID-19 infection [10,11,12].

However, previous research has shown that the wide range of ongoing symptoms does not always align with cardiovascular dissociation or myocardial injury [7]. It is crucial to identify the specific pathophysiological mechanisms of the cardiovascular manifestation of long COVID using appropriate imaging techniques to gain a better understanding of the condition and develop effective treatment strategies. The impact of viral infection on the cardiovascular system can increase cardiovascular morbidity and mortality [13]. Valuable diagnostic methods such as laboratory analyses and clinical examination and multimodality imaging methods such as echocardiography, cardiac magnetic resonance (CMR), and computer tomography may be utilized to find cardiovascular manifestations in these conditions. However, some delayed complications remain not fully understood. The studies demonstrated subtle consistent reductions in left ventricle systolic and diastolic function as well as right ventricle functions using conventional and advanced 2D speckle-tracking echocardiography [14,15,16]. The findings highlight the need for cardiac assessments in long-term follow-up and the potential impact on clinical outcomes [17].

This study aimed to investigate the mechanisms that contribute to changes in cardiac structure, inflammation, and fibrosis, as well as the impacts on both systolic and diastolic cardiac function. We utilized standard and advanced clinical and imaging assessment methods, including echocardiography and cardiac magnetic resonance (CMR) imaging. The goal was to effectively identify a group of high-risk patients and ensure they receive appropriate therapy.

## 2. Materials and Methods

This prospective observational study was conducted at the University Clinical Centre of Serbia (UCCS) from January to July 2021. These patients were treated at the Infectious and Tropical Diseases Clinic of the University Clinical Center of Serbia for acute COVID-19 infection, and some had been hospitalized.

We screened consecutive outpatient convalescents from COVID-19 who were in follow-up care and presented to the Cardiology Department with symptoms indicative of long COVID. These patients reported a variety of symptoms, with the duration ranging from 1 month to 14 months. The average observation period occurred 6.2 ± 2.7 months after the onset of the COVID-19 infection.

**Figure 1:**
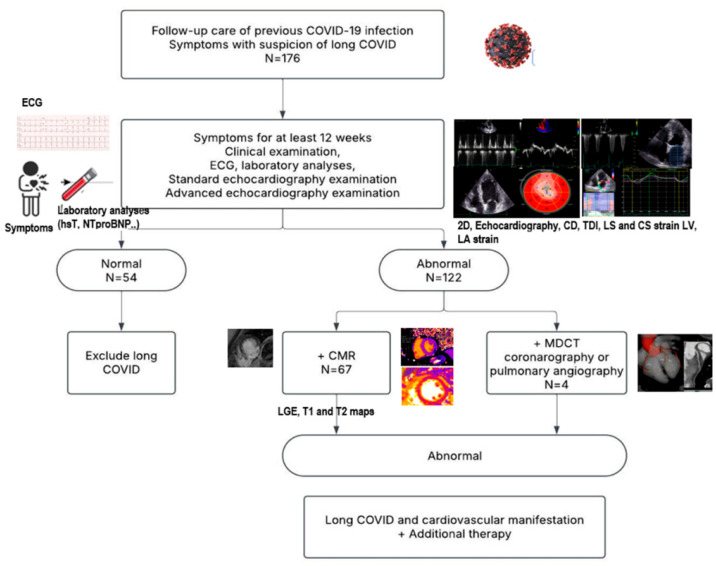
Protocol flowchart.

The inclusion criteria were the persistence of symptoms such as effort intolerance and/or fatigue and shortness of breath not explained by any other illness, age > 18 years, signed informed consent stating their willingness to participate, and previous positive nasopharyngeal and oropharyngeal PCR test for COVID-19. The criteria for diagnosing long COVID were persisting symptoms such as effort intolerance and/or fatigue and shortness of breath for at least 12 weeks following an acute COVID-19 infection (Figure 1). We used additional diagnostic and imaging methods to identify cardiovascular manifestations of long COVID. The patients underwent clinical examination, laboratory analysis, and conventional and advanced imaging echocardiography. In a group of patients without symptoms of adequate duration and no pathological findings, long COVID was excluded. The cardiac magnetic resonance (CMR), coronary computed tomography angiography (CCTA), and pulmonary angiography (PA) were performed in accordance with local regulative rules for patients with suspicion of long COVID diagnosis (Figure 1). CMR was performed on patients who met the criteria related to the duration of symptoms, as well as abnormalities identified in their electrocardiograms (ECGs) and echocardiograms. The following conditions were contraindications for the CMR procedure: the presence of metallic fragments (such as bullets, shrapnel, or pellets), cerebral aneurysm clips, magnetic dental implants, tissue expanders, prosthetic limbs, hearing aids, body piercings, claustrophobia, and the patient’s failure to sign a consent form for the procedure.

The exclusion criteria were a history of significant heart failure and/or reduced ejection fraction (<50%), persistent hypertension, significant ischemic disease, arrhythmia as well as previous significant pulmonary disease. Diseases such as pulmonary, gastrointestinal, hematological, and nephrological conditions were found in the differential diagnosis.

National and international ethical guidelines were followed during this study with approval obtained from the Ethics Committee of the University Clinical Centre of Serbia and as part of the Project of the Science Fund of the Republic of Serbia named “AntioxIdentification” (No 7546803) [18].

All the echocardiographic examinations were performed using Vivid E95 (General Electric, UK and Echo Pack PC version 203 for offline analyses) with a 3.5 MHz transducer in the parasternal (long- and short-axis views) and apical views (two- and four-chamber and apical long-axis views). All definitions and rules for measurements were in accordance with the recommendations of the American Society of Echocardiography (ASA) and the European Association of Cardiovascular Imaging (EACVI) [19,20,21,22,23].

All patients underwent a comprehensive echocardiographic examination and advanced echocardiography using two-dimensional (2D) speckle-tracking echocardiography, which permits the assessment of myocardial deformation. The advanced echocardiography methods were utilized to better understand the mechanisms of cardiovascular disorders associated with long COVID. We analyzed longitudinal strain (LS), which represents the myocardial shortening in the long-axis plane, and circumferential strain (CS), which represents myocardial shortening in the short-axis plane, for all subjects. Moreover, LS and CS were calculated through the three myocardium layers. We defined the following three layers: sub-epicardium (epi), intra-myocardium (mid), and sub-endocardium (endo). Six myocardial walls and 18 segments in the longitudinal and short-axis views (three slices—base, mid, and apex) were analyzed. In end systole, a semi-automated function defined a region of interest (ROI) to assist in tracing the endocardial border. The investigator visually evaluated the ROI to ensure that the tracking of speckles and their widths was accurate, making manual adjustments when necessary. All findings were analyzed and measured offline in Echo Pac PC.

The CMR examinations were performed in a group of patients with a diagnosis of long COVID and cardiovascular manifestation. CMR was performed on a clinical 1.5-T scanner (Simens Avanto, Forchheim, Germany), using standardized and unified imaging protocols (University Clinical Center of Serbia, Center of CMR, Belgrade, Serbia). CMR protocols for the assessment of morphological and functional characteristics, including late gadolinium enhancement (LGE) and T1 and T2 mapping using the MOLLI sequence, were performed [24]. CMR analyses were extracted offline via Syngo.via software (Siemens Healthineers, Forchheim, Germany), using a manual adjustment of the ventricular contours applied to determine the LV mass, end-diastolic volumes, and ejection fractions. Myocardial T1 and T2 mapping were determined as regions of interest (ROIs) where the highest values of T1 and T2 were found, pre- and post-contrast, in LV myocardium segments. The myocardial extracellular volume (ECV), expressed as % myocardium volume, was conventionally computed from: (i) T1-pre values from the pre-contrast MOLLI sequence; (ii) T1- post values from post-contrast MOLLI sequence (acquisition scheme: 4(1)3(1)2) acquired 10–15 min after the injection; and (iii) individual hematocrit values were obtained from blood sampled just before CMR. The formula for ECV (%) is: 100% × (1-hematocrit) × [(1/post-contrast T1 myocardium) − (1/nativeT1myocardium)]/[(1/post-contrast T1 blood) − (1/nativeT1 blood)].

Continuous variables were expressed as the mean values with corresponding standard deviations for normally distributed data. Categorical variables were expressed as frequencies and percentages. The parameters of the two subgroups were compared using Student’s t-test or the Mann–Whitney U test, depending on the data distribution. Proportional differences were evaluated with a chi-squared test. The associations of clinical and imaging parameters were analyzed by means of linear logistic regression models for univariate and multivariate analyses. Variables with a univariate value of *p* < 0.05 were incorporated into the multivariate analysis. A Pearson’s correlation was used to find the linear association between variables of two imaging methods. The IBM SPSS Statistics 26.0 software was used in the analyses.

## 3. Results

### 3.1. Clinical and Laboratory Indicators of Long COVID

In this study, 176 pts with previous COVID-19 infection, 55.9 ± 12.3 years of age, 58.5% males, 84.7% hospitalized, and 1.7% mechanically ventilated, came to cardiologist follow-up care.

The dominant symptoms of patients with suspicion of long COVID were effort intolerance and/or fatigue and shortness of breath. In addition to these symptoms, palpitation, irregular pulse or premature ventricular beats (PVBs), and tightness of the chest were also present in the analyzed patients (Table 1). However, there were no differences observed in the occurrence of arrhythmias between the two groups.

Based on the criteria for diagnosis of long COVID, patients were divided into two groups: patients with long COVID and cardiovascular manifestation (*n* = 122) and those without long COVID (*n* = 54).

Most patients diagnosed with long COVID were hospitalized during the acute phase of COVID-19 and had longer hospital stays compared to those without long COVID. The results also indicated that a majority of patients with long COVID developed hypertension during their illness and showed slightly lower hemoglobin levels in laboratory analyses (Table 1). The other risk factors and associated comorbidities were not significantly different between the two groups.

### 3.2. Cardiac Structure and Function Echocardiographic Parameters Suggesting Long COVID

Patients with long COVID had significantly lower values of left ventricular ejection fraction (LVEF) (Table 2).

The parameters of diastolic function of the left ventricle (LV), such as e’ lateral and left atrial reservoir strain, were significantly lower in the group of patients with long COVID (Table 2).

Global LS and the peak systolic strain of the endo and mid layers were significantly lower in long COVID patients compared with the other group of patients (Table 2).

**Figure 2:**
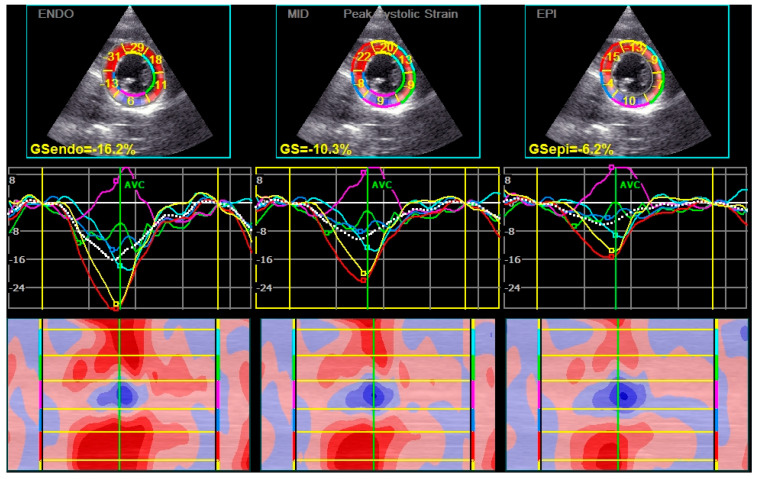
The levels of CS in patients with Long COVID were decreased. The lowest levels of CS were found in the epicardial layers. In this patient, the values in the inferior segments of the left ventricle were particularly changed (pink color).

Peak systolic CS of the epi layer was significantly lower in patients with long COVID (Table 1) (Figure 2). The post-systolic shortening of the mid and epi layers of CS was also significantly lower in the long COVID group of patients (Table 2).

The univariate analysis showed that the potential echocardiographic parameters of long COVID were LVEF, peak systolic CS of the epi layer, and post-systolic circumferential shortening in the epi and mid layers (Table 3). The cut off value of EF was 54.5%, with high sensitivity (Sen 88.4%, *p* = 0.002); the cut off value of peak systolic CS of the epi layer was −9.97% (Sen 70.4%, *p* = 0.021); the cut off value of CS post-systolic shortening in the mid layer was 0.0813 s (Sen 63%, *p* = 0.006); and in the epi layer, it was 0.101 s (Sen 74%, *p* = 0.001).

### 3.3. Cardiac Structure CMR Parameters Suggesting Long COVID

**Figure 3:**
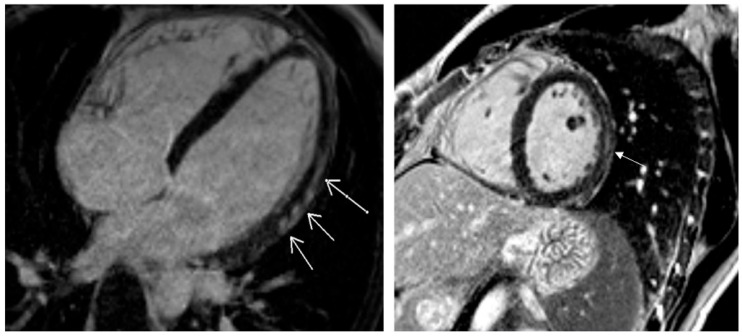
Post-contrast findings of late gadolinium enhancement localized in sub-epicardial and mid-myocardial layers (white arrows).

**Figure 4:**
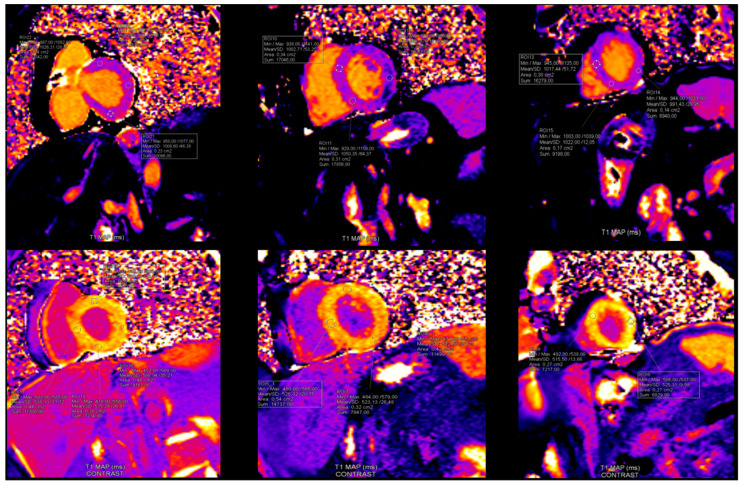
Findings in native and post-contrast T1 mapping. In this patient with Long COVID: T1 native maps were normal, however T1 postcontrast maps were increased in anteroseptal segments in basal and mid level and in all segments in apical level of left ventricle. (Reference rate: base 1058.7 ± 53.7 ms, mid 1025.8 ± 36.9 ms, apex 1048.9 ± 57.1 ms) (reference rate: base 408.2 ± 53.6 ms, mid 420.5 ± 50.8 ms, apex 396.1 ± 48.3 ms).

**Figure 5:**
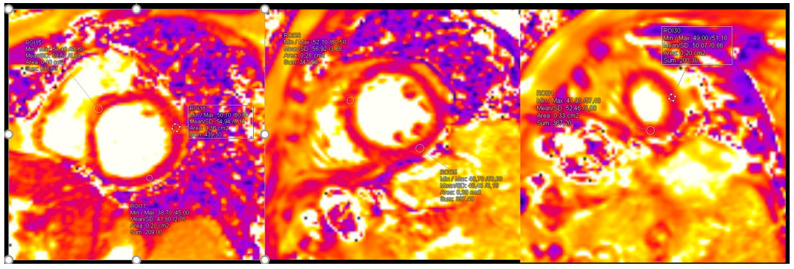
Pathological findings in native T2 mapping (reference rate: base 48.1 ± 3.3 ms, mid 47.4 ± 3.2 ms, and apex 51.3 ± 4.2 ms).

Pathological CMR examinations in the group of patients with long COVID were seen in 58.2% of patients. LGE was positive in 45.3% of patients (Table 4). The distribution of LGE was predominantly in the intramyocardial and sub-epicardial layers of the myocardium (Figure 3). Distribution in ≥3 segments in accordance with the American Heart Association was identified in 37.3% [18] (Table 4). Pathological findings in native T1 mapping were discovered in 21.0% of patients (Figure 4), and it was a similar percentage in post-contrast T1 mapping (19.4%). Pathological findings in native T2 mapping were observed in 22.4% of patients (Figure 5) (Table 4). In the group of patients with increased native T1 mapping, 50% showed LGE positivity. Meanwhile, among patients with increased post-contrast T1 mapping, 37.3% were LGE positive. Additionally, 70% of patients with increased native T2 mapping were positive for LGE. Pericarditis was found in 12.5% of patients with long COVID.

**Figure 6:**
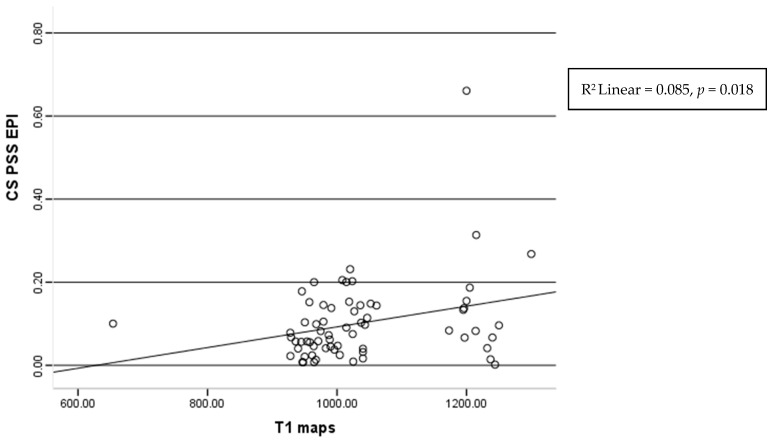
Correlation of the T1 maps and post-systolic shortening of epicardium layers of circumferential strain.

A significant correlation between the post-systolic shortening values in the epi circumferential layer and the native T1 mapping values was found (r = 0.212, *p* = 0.018) (Figure 6).

## 4. Discussion

Long COVID is an unpredictable condition that presents a range of symptoms affecting multiple organ systems. In this study, we identify a few potential mechanisms of changing cardiac structure and function in long COVID by utilizing multimodality imaging.

### 4.1. Clinical Presentations and Laboratory Markers of Cardiovascular Manifestation

In our research, cardiovascular manifestations align with previous studies, which found that the dominant cardiovascular symptoms include shortness of breath, effort intolerance or fatigue, palpitations, irregular pulse or premature ventricular beats (PVBs), and chest tightness. However, the mechanisms underlying the cardiovascular issues of long COVID are not always fully understood, as previously highlighted [25]. Our results showed a significant difference in the incidence of long COVID-19 between patients who were hospitalized during their acute infection and those who were not. A large cohort study indicating a strong correlation between the severity of illness, the length of hospital stays, and higher rates of long COVID-19 symptoms was reported among previously hospitalized patients [26,27,28,29]. Consequently, the relationship may be attributed to the severity of inflammation and the complexity of the immunological process, which could lead to the pathological cardiovascular findings observed in these patients.

Additionally, we found that a higher percentage of individuals developing hypertension during COVID-19 infections may contribute to the onset of long COVID in this patient group. The hypertension in long COVID may be linked to pathological disorders, including those related to the kidneys, cerebrovascular issues, and endocrine diseases [30].

The phenotype of patients with decreased levels of hemoglobin was noticed in the group with long COVID. This finding is aligned with previous studies and findings that iron dysmetabolism present in COVID patients could potentially lead to restricted hemoglobin synthesis, leading to anemia and sustained hypoxia in long COVID [31,32]. Moreover, recently, this hemoglobin impairment was connected as an important player to the fatigue development experienced by these patients [33].

Markers of inflammation and myocardial necrosis may increase during the post-acute phase of COVID-19 and persist into the follow-up period after discharge. The levels of these markers could correlate with complications experienced by patients with long COVID [34,35,36]. The changes in the myocardium can be attributed to damage by direct viral tropism, cytotoxic viral proteins, and immune system activation, which can lead to an autoimmune response or prolonged, nonspecific inflammation, causing further harm to host cells [37]. The findings regarding the genome polymorphisms on inflammation and coagulation markers could have important clinical implications, and the results may enable a more personalized approach for identifying patients at a higher risk of cardiac dysfunction or thrombosis and for determining the need for targeted antioxidant therapy [18,38,39,40].

While cardiovascular involvement in long COVID may be asymptomatic, individuals who develop symptoms over time are more likely to experience abnormal findings detected through imaging techniques such as CMR or advanced echocardiography [41,42,43,44].

### 4.2. Multimodality Imaging Parameters in Long COVID Cardiovascular Manifestations

The imaging findings that present cardiac damage may be useful to specify the mechanism in long COVID patients. We aimed to identify the specific cardiovascular pathophysiological mechanism utilizing the imaging methods and provide accurate diagnoses and treatment of patients suspicious of long COVID-19. Diagnosing myocardial alterations resembling “ischemia”, “myocarditis”, or “heart failure” in this group of patients is challenging and requires a combination of advanced imaging techniques and clinical observations. It seems that persistent inflammation following COVID-19 infection can lead to either of these pathways [45]. CMR is considered the most effective way to assess myocardial tissue pathology, particularly myocardial edema, inflammation, and fibrosis, which cannot be precisely detected using other imaging techniques [45].

CMR with late gadolinium enhancement (LGE) can reveal myocardial injury and replacement fibrosis, which may be of ischemic or inflammatory origin. Elevated CMR T1 mapping may indicate diffuse fibrosis or infiltration, while elevated CMR T2 mapping may often indicate diffuse myocardial edema [36,41,42,43].

Nevertheless, CMR may not always be available in medical centers. Echocardiography could be the first diagnostic method for identifying structural changes before any other signs or symptoms of heart disease, after recovering from COVID-19 [46,47,48]. Monitoring several conventional echocardiographic parameters, such as LVEF, can be beneficial, as we pointed out. Moreover, it is important to note that some patients with symptoms of dyspnea and signs of heart failure may have normal or even supra-normal LVEF, indicating that LVEF alone is not sufficient for the assessment of these patients [49]. It is important to understand that the LVEF can be affected by various diseases and that determining its value alone does not provide insight into a specific pathophysiological mechanism. In patients who have recovered from COVID-19, a decreased ejection fraction may result from a few primary mechanisms: prolonged inflammation associated with myocarditis-like conditions, early-stage heart failure, and issues related to microvascular disease and myocardial ischemia. However, a preserved ejection fraction is more common among long COVID patients, except in those who are in advanced stages of heart failure [48].

Echocardiographic assessment of left ventricular diastolic function is also an integral part of the routine evaluation of patients presenting with dyspnea [49]. We observed parameters of left ventricular diastolic function and found a decrease in the e’ lateral wave measured by tissue Doppler, as well as reduced left atrial reservoir strain assessed by 2D speckle-tracking strain analysis, in a group of patients with long COVID. These findings may indicate that a group of patients is developing early-stage heart failure or an early pre-ischemic state. New speckle-tracking strain parameters analyzed by myocardial layers, along with CMR, facilitate early disease diagnosis. Strain echocardiography, which assesses the myocardial deformation in two dimensions and differentiates between conditions, is more cost-effective and can be performed at the patient’s bedside [50,51,52,53,54,55]. Global LS of the left and right ventricle can be independent predictors of in-hospital mortality in patients with COVID-19 [52]. Damage to the subendocardial longitudinal fibers may present as pre-ischemic disease, and it could be found using LS [54]. In accordance with previous studies, we analyzed the LS and CS in three layers and suggest that patients with long COVID may exhibit disorders similar to ischemic heart disease, even in the absence of significant stenosis in the coronary arteries, possibly due to temporary blood thrombosis or micro-embolism. In previous studies, global LS was reduced (<−21.0%) in about 83% of COVID-19 patients, and the alteration was more prominent in the subepicardium than in the subendocardium [46]. Similarly, global LS, as well as the peak systolic strain of the endo and mid layers, was significantly lower in long COVID patients in our study. The changes in the CS epi layer may indicate that changes may occur in patients with possible myocarditis. These specific changes in CS may be associated with edema or fibrosis in the epicardium and myocardial middle layers of the left ventricle [52]. In our study, the peak systolic CS in the epi layers and circumferential post-systolic shortening in epi and mid layers were significantly decreased in long COVID patients. We identified a significant correlation between post-systolic shortening in the epicardial circumferential layers and native T1 mapping.

In our study, patients who presented with high suggestion of long COVID after abnormal clinical and echocardiographic examination had 58.2% abnormal CMR findings. Among these, 43.3% exhibited positive late gadolinium enhancement (LGE) classified as “inflammatory” or another “non-ischemic” type. Myocardial injury and replacement fibrosis were primarily located in the subepicardial region (37.9%), both the subepicardial and intramuscular regions (41.4%), or solely in the intramuscular regions (20.7%). We did not observe any transmural or subendocardial LGE deposition. In the study conducted by Kotecha T, et al. [36], a “myocarditis-like” or “inflammatory” scar pattern, characterized by sub-epicardial LGE on CMR, was found in 26% of the patients. Additionally, an infarction and/or ischemia scar pattern, which is characterized by sub-endocardial or transmural LGE on CMR, was found in 22% of the patients. Finally, dual pathology was observed in 6% of the patients. In our study, significant fibrosis was observed in 37.3% of patients, with fibrosis present in three or more segments, according to the standardized myocardial segmentation defined by the American Heart Association [19]. We also found increased values of native T1, indicating persistent inflammation and potential interstitial fibrosis, in 21.0% of patients. Elevated native T2 mapping, indicating ongoing inflammation and possible edema, was observed in 22.4% of patients. Among those with increased native T1 values, 50% had negative LGE, while 30% of patients with elevated native T2 values also had negative LGE. These patients had only inflammation and/or edema, without manifested fibrosis. In previous studies, findings of pathological CMR characterized by abnormal LGE or native T1 or native T2 ranged from 37 to 75% [42,45].

**Figure 7:**
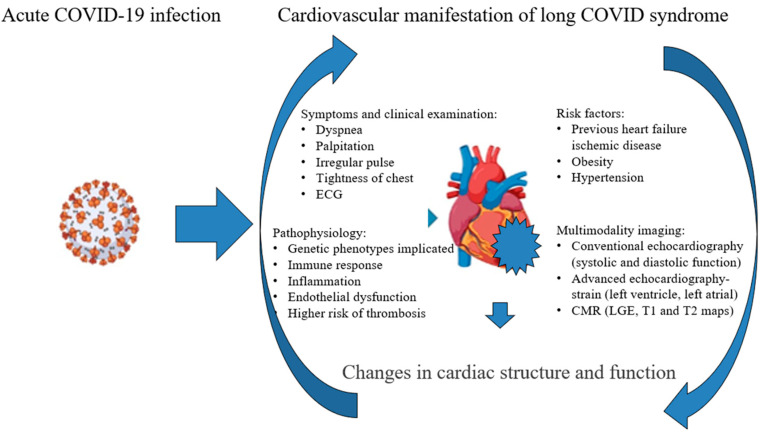
Changes in cardiac structure and function—potential mechanisms.

To address the complexities of long COVID, we must prioritize research into its underlying mechanisms and the specific processes that drive its symptoms. Additionally, it is essential to recognize that pre-existing conditions and potential genetic factors significantly determine why some individuals experience lingering symptoms [56]. The role of the immune response is crucial in understanding long COVID syndrome; however, we need to delve deeper into that mechanism through future research (Figure 7).

In Figure 7, we highlight the complexity of the timely detection of changes in cardiac structure and function. This process involves a comprehensive approach that includes clinical examinations, assessing risk factors, and identifying potential pathophysiological mechanisms through echocardiography. Advanced echocardiography techniques and cardiac magnetic resonance (CMR) imaging are also critical in this evaluation. It is essential to consider potential therapies that target the underlying pathophysiological processes. Treatment options should encompass anti-inflammatory medications, antithrombotic or anticoagulant therapy, antiarrhythmic drugs, and therapies specifically designed for the early stages of heart failure.

### 4.3. Limitations

The limitation factors are the number of analyzed patients, the lack of randomization, and the short-term follow-up. Another limitation is the single-center research design. In addition, CMR could not be conducted due to technical challenges and predefined contraindications in certain patient populations.

## 5. Conclusions

Most patients with long-COVID showed structural and functional changes in their cardiovascular systems, primarily caused by prolonged inflammation. Using multimodality imaging, such as echocardiography and CMR, especially, is crucial. This multimodality imaging technique is essential to find the mechanisms of predicting chronic myocarditis, early-stage heart failure, and pre-ischemic states, which can lead to serious complications. Recognizing the specific cardiovascular phenotypes associated with long COVID is essential to provide timely and appropriate treatment.

## Figures and Tables

**Table 1:** Overall data of patients with suspicion of long COVID and cardiovascular manifestation.

Baseline Characteristics	Total (*n* = 176)	Long COVID		*p*
		No (*n* = 54)	Yes (*n* = 122)	
Age, yr (mean ± SD)	55.9 ± 12.3	56.07 ± 12.41	55.89 ± 12.24	0.453
Sex m/f, *n* (%)	103/73 (58.5/41.5)	34/20 (63/37)	69/53 (56.6/43.4)	0.426
Hospitalized, *n* (%)	149 (84.7)	41 (75.9)	108 (88.5)	0.032
Length of hospital stay median (25th–75th percentile)	12.37 ± 6.86	10.59 ± 4.14	13.11 ± 7.54	0.044
Non-invasive mechanical ventilation (NIV), *n*, (%)	1 (0.6)	0 (0)	1 (0.9)	1.0
Mechanically ventilation, *n* (%)	1 (0.6)	0 (0)	1 (0.9)	1.0
BMI (kg/m^2^) (mean ± SD)	28.9 ± 4.9	28.31 ± 4.73	29.22 ± 4.96	0.060
SBP (mmHg) (mean ± SD)	128.3 ± 19.5	130.93 ± 18.98	127.17 ± 19.65	0.239
DBP (mmHg) (mean ± SD)	78.5 ± 11.1	80.46 ± 11.59	77.66 ± 10.89	0.125
Patient symptoms and clinical signs				
Irregular pulse or PVB, *n* (%)	67 (38.1)	16 (29.6)	51 (41.8)	0.125
Palpitation or tachycardia, *n* (%)	60 (34.1)	15 (27.8)	45 (36.9)	0.240
Tightness of chest, *n* (%)	59 (33.5)	16 (29.6)	43 (35.2)	0.467
Risk factors				
Obese status				0.052
Obesity, *n* (%)	62 (35.2)	13 (24.1)	49 (40.2)	
Pre-obesity, *n* (%)	81 (46.0)	32 (59.3)	49 (40.2)	
Non-obese, *n* (%)	33 (18.8)	9 (16.7)	24 (19.7)	
Smoking status				0.861
Current, *n* (%)	23 (13.1)	7 (13)	16 (13.1)	
Previous, *n* (%)	57 (32.4)	16 (29.6)	41 (33.6)	
Never, *n* (%)	96 (54.5)	31 (57.4)	65 (53.3)	
Hypertension, *n* (%)	81 (46.0)	18 (33.3)	63 (51.6)	0.025
Previous myocardial infarction, *n* (%)	7 (4.0)	1 (1.9)	6 (4.9)	0.678
Previous stroke, *n* (%)	3 (1.7)	2 (3.7)	1 (0.8)	0.223
Previous heart failure (*n*, %)	0 (0.0)			
Chronic obstructive pulmonary disease, *n* (%)	10 (5.7)	2 (3.7)	8 (6.6)	0.726
Diabetes, *n* (%)	29 (16.5)	8 (14.8)	21 (17.2)	0.692
Malignancy, *n* (%)	10 (5.7)	2 (3.7)	8 (6.6)	0.726
Laboratory analysis				
Hs Troponin T (ng/mL) (mean ± SD)	8.95 ± 12.8	7.63 ± 4.33	9.51 ± 14.97	0.377
BNP (pg/mL), median (25th–75th percentile)	18 (10–39)	17 (10–29)	18 (11–44)	0.333
D dimer, median (25th–75th percentile)	0.34 (0.25–0.54)	0.32 (0.23–0.50)	0.35 (0.26–0.60)	0.181
Ferritin (g/L), median (25th–75th percentile)	134.6 (78.8–230.8)	149.5 (67.85–213.48)	132.8 (86–238.5)	0.824
Leucocytes (×10^9^) (mean ± SD)	6.7 ± 1.8	6.40 ± 1.44	6.89 ± 1.88	0.090
Haemoglobin (g/L) (mean ± SD)	142 ± 12.8	145.46 ± 14.21	141.16 ± 12.02	0.040
Hs CRP, median (25th–75th percentile)	1.55(0.8–3.55)	1.35 (0.8–3.33)	2.0 (0.88–3.63)	0.258

Legend: BMI—Body Mass Index; SBP—Systolic Blood Pressure; DBP—Diastolic Blood Pressure; PVB—Premature Ventricular Beat; BNP—B-type Natriuretic Peptide; Hs CRP—High-sensitivity C-Reactive Peptide.

**Table 2:** Echocardiographic parameters of patients with long COVID and cardiovascular manifestations.

	Long COVID, Yes *n* = 122	Long COVID, No *n* = 54	*p*
Conventional echocardiography LV parameters			
LVM (g) mean ± SD	177.1 ± 51.3	176.9 ± 47.8	0.380
LVMI (g/m^2^) mean ± SD	84.6 ± 21.3	86.1 ± 20.4	0.854
LVEDV (mL) mean ± SD	108.5 ± 29.9	109.8 ± 31.5	0.810
LVEDVI (mL m^2^) mean ± SD	55.8 ± 47.3	53.2 ± 12.1	0.681
LVESV (mL) mean ± SD	50.6 ± 17.6	48.3 ± 14.6	0.363
LVESVI (mL m^2^) mean ± SD	24.2 ± 7.5	23.7 ± 7.3	0.720
LVEF (%) mean ± SD	59.6 ± 5.5	62.3 ± 3.6	0.001
E (m/s) mean ± SD	0.59 ± 0.16	0.57 ± 0.10	0.538
E/A mean ± SD	0.92 ± 0.30	0.91 ± 0.25	0.820
e’ septal (m/s) mean ± SD	0.08 ± 0.06	0.07 ± 0.03	0.626
e’ lateral (m/s) mean ± SD	0.09 ± 0.03	0.10 ± 0.06	0.038
E/e’ mean ± SD	7.7 ± 2.4	7.3 ± 1.9	0.273
DT (ms) mean ± SD	214.2 ± 47.5	211.5 ± 54.6	0.753
RVSP (mmHg) mean ± SD	26.97 ± 6.26	26.57 ± 5.16	0.807
Diastolic dysfunction, *n* (%)	62 (50.8)	28 (51.85)	0.488
Grade of diastolic dysfunction, *n* (%)			
1	58 (47.5)	27 (50)	0.486
2	6 (4.9)	1 (1.85)	
3	0	0	
Echocardiographic signs of edema, *n* (%)	33 (27.1)	20 (37)	0.272
Echocardiographic signs of fibrosis, *n* (%)	50 (41)	17 (31.5)	0.050
Advanced echocardiography LV parameters			
GLS endo (%) mean ± SD	−21.5 ± 2.9	−22.5 ± 3.1	0.041
GLS mid (%) mean ± SD	−19.1 ± 2.5	−19.9 ± 2.7	0.048
GLS epi (%) mean ± SD	−17.3 ± 2.3	−17.9 ± 2.4	0.064
Peak Systolic LS endo (%) mean ± SD	−20.9 ± 3.1	−22.0 ± 3.2	0.038
Peak Systolic LS mid (%) mean ± SD	−18.6 ± 2.7	−19.5 ± 2.7	0.043
Peak Systolic LS epi (%) mean ± SD	−16.8 ± 2.4	−17.5 ± 2.5	0.056
GLSr S (1/s) mean ± SD	−0.98 ± 0.15	−1.03 ± 0.18	0.172
GLSr E (1/s) mean ± SD	1.07 ± 0.29	1.06 ± 0.28	0.747
GLSr A (1/s) mean ± SD	0.93 ± 0.22	0.97 ± 0.21	0.147
LS Post-systolic shortening endo (s) mean ± SD	0.032 ± 0.031	0.027 ± 0.020	0.301
LS Post-systolic shortening mid (s) mean ± SD	0.034 ± 0.031	0.029 ± 0.022	0.328
LS Post-systolic shortening epi (s) mean ± SD	0.036 ± 0.031	0.031 ± 0.024	0.357
Peak Systolic CS endo (%) mean ± SD	−23.7 ± 4.2	−24.7 ± 4.6	0.183
Peak Systolic CS mid (%) mean ± SD	−15.4 ± 2.8	−16.4 ± 3.2	0.056
Peak Systolic CS epi (%) mean ± SD	−9.6 ± 2.3	−10.5 ± 2.6	0.029
CS Post-systolic shortening endo (s) mean ± SD	0.086 ± 0.059	0.071 ± 0.056	0.093
CS Post-systolic shortening mid (s) mean ± SD	0.101 ± 0.069	0.075 ± 0.055	0.006
CS Post-systolic shortening epi (s) mean ± SD	0.138 ± 0.111	0.088 ± 0.064	<0.001
Torsion (°/cm) mean ± SD	1.96 ± 0.72	2.02 ± 0.78	0.621
Twist (°) mean ± SD	15.03 ± 5.11	15.30 ± 5.63	0.749
Time to peak twist (ms) mean ± SD	345.27 ± 52.63	328.71 ± 54.20	0.053
Peak rotation apex (°) mean ± SD	7.99 ± 3.72	8.17 ± 3.99	0.774
Time to peak rotation apex (ms) mean ± SD	352.30 ± 117.63	359.08 ± 98.98	0.696
Peak rotation base (°) mean ± SD	−6.68 ± 3.71	−7.03 ± 4.17	0.594
Time to peak rotation base (ms) mean ± SD	407.47 ± 167.08	368.59 ± 122.61	0.115
Conventional and advanced echocardiography RV parameters			
RVEF (%) mean ± SD	60.2 ± 10.1	61.0 ± 11.4	0.604
RV FAC (%) mean ± SD	45.4 ± 8.8	46.2 ± 10.1	0.613
RV GLS (%) mean ± SD	−23.1 ± 6.4	−23.4 ± 5.3	0.791
Sa (cm/s) mean ± SD	0.13 ± 0.03	0.12 ± 0.03	0.510
Conventional echocardiography LA parameters			
LAV (mL) mean ± SD	53.4 ± 13.7	50.9 ± 13.2	0.259
LAVI (mL/m^2^) mean ± SD	26.8 ± 7.5	24.9 ± 6.4	0.099
Advanced echocardiography LA parameters			
LAs R (%) mean ± SD	24.85 ± 5.96	27.22 ± 8.41	0.034
Time to peak LAs R (ms) mean ± SD	427.99 ± 53.21	399.79 ± 64.46	0.003
LAs CD (%) mean ± SD	−13.06 ± 4.77	−13.45 ± 5.32	0.622
LAs pump (%) mean ± SD	−12.36 ± 3.89	−13.28 ± 5.05	0.194
Time to peak LAs pump (ms) mean ± SD	741.34 ± 113.22	732.46 ± 108.04	0.627
LAsr S (1/s) mean ± SD	1.19 ± 0.75	1.23 ± 0.35	0.708
Time to peak LAsr S (ms) mean ± SD	178.17 ± 71.11	154.91 ± 50.55	0.031
LAsr E (1/s) mean ± SD	−1.08 ± 0.46	−1.06 ± 0.47	0.662
Time to peak LAsr E (ms) mean ± SD	526.90 ± 55.31	497.98 ± 75.63	0.005
LAsr A (1/s) mean ± SD	−1.47 ± 1.27	−1.61 ± 0.62	0.439
Time to peak LAsr A (ms) mean ± SD	832.05 ± 147.14	808.98 ± 120.59	0.313

Legend: CS—Circumferential Strain; DT—Deceleration Time; E—early diastolic filling; A—atrial diastolic filling; E/A ratio—ratio of early and late diastolic inflow velocities; e’ septal—septal mitral anulus velocity; e’ lateral—lateral mitral anulus velocity; E/e’ ratio—ratio of early diastolic inflow velocity and early diastolic movement of the mitral annulus; GLS—Global Longitudinal Strain; LAV—Left Atrial Volume; LAVI—Left Atrial Volume Index; LAs R—Left Atrial strain Reservoir; LAs CD—Left Atrial strain Conduit; LAsr—Left Atrial strain rate (S wave, E wave, A wave); LVEDV—Left Ventricle End-Diastolic Volume; LVEDVI—Left Ventricle End-Diastolic Volume Index; LVESV—Left Ventricle End-Systolic Volume; LVESVI—Left Ventricle End-Systolic Volume Index; LVEF—Left Ventricle Ejection Fraction; LVM—Left Ventricle Mass; LVMI—Left Ventricle Mass Index; LS—Longitudinal Strain; RVEF—Right Ventricle Ejection Fraction; RV FAC—Right Ventricle Fractional Area Change; RV GLS—Right Ventricle Global Longitudinal Strain; RVSP—Right Ventricle Systolic Pressure; Sa—Tricuspid Annular Systolic Velocity.

**Table 3:** Conventional and advanced echocardiographic parameters of long COVID.

Variable	Univariate Analysis OR (95% CI)	*p* Value
LVEF	0.023 (0.010–0.036)	0.001
GLSendo	0.014 (0.009–0.037)	0.220
GLSmid	0.014 (0.011–0.040)	0.269
Peak Systolic LS endo	1.396 (1.061–3.854)	0.264
Peak Systolic LS mid	1.276 (1.161–3.712)	0.303
Peak Systolic CS epi	0.028 (0.001–0.055)	0.041
CS Post-systolic shortening endo	0.855 (0.350–2.060)	0.163
CS Post-systolic shortening mid	1.420 (0.309–2.530)	0.013
CS Post-systolic shortening epi	1.489 (0.711–2.267)	<0.001
LAs reservoir	0.011 (0.001–0.021)	0.034

Legend: LVEF—Left Ventricle Ejection Fraction; GLS—Global Longitudinal Strain; LS Longitudinal Strain; CS—Circumferential Strain; LAs—Left Atrial Strain.

**Table 4:** Cardiac magnetic resonance parameters of patients with long COVID and cardiovascular manifestations.

CMR Parameters	Long COVID
	*n* = 67
LVEDV, mL (mean ± SD)	126.71 ± 28.83
LVEDVI, mL/m^2^ (mean ± SD)	64.26 ± 12.87
LVESV, mL (mean ± SD)	46.52 ± 15.37
LVESVI, mL/m^2^ (mean ± SD)	24.14 ± 6.54
LVEF, % (mean ± SD)	61.97 ± 6.02
LVSV, mL (mean ± SD)	77.78 ± 16.22
LVSVI, mL/m^2^ (mean ± SD)	39.79 ± 7,93
LVM, g (mean ± SD)	116.24 ± 36.56
LVCI, L/min/m^2^ (mean ± SD)	2.71 ± 0.63
RVEDV, mL (mean ± SD)	125.34 ± 32.84
RVEDVI, mL/m^2^ (mean ± SD)	62.97 ± 14.36
RVESV, mL (mean ± SD)	49.96 ± 16.80
RVESVI, mL/m^2^ (mean ± SD)	24.97 ± 7.03
RVEF, % (mean ± SD)	61.64 ± 6.01
RVSV, mL (mean ± SD)	74.69 ± 17.88
RVSVI, mL/m^2^ (mean ± SD)	39.38 ± 8.06
RVCI, L/min/m^2^ (mean ± SD)	2.65 ± 0.58
T1 native, ms (mean ± SD)	1035.94 ± 111.43
T1 native increase, *n* (%)	14 (21.0)
T1 post-contrast, ms (mean ± SD)	440.81 ± 99.55
T1 post-contrast increase, *n* (%)	13 (19.4)
T2 native, ms (mean ± SD)	45.21 ± 3.05
T2 post-contrast increase, *n* (%)	15 (22.4)
LGE positive, *n* (%)	29 (43.3)
LGE in ≥3 segments, *n* (%)	25 (37.3)
LGE layers, *n* (%)	
epi	11 (37.9)
epi or mid	12 (41.4)
mid	6 (20.7)
Total abnormal, *n* (%)	39 (58.2)
LGE positive in T1 native increase patients (%)	50.0
LGE positive in T1 post-contrast increase patients (%)	30.8
LGE positive in T2 native increase patients (%)	70.0
Pericarditis, *n* (%)	8 (12.5)

Legend: LGE—Late Gadolinium Enhancement, LVCI—Left Ventricle Cardiac Index, LVEDV—Left Ventricle End-Diastolic Volume, LVEDVI—Left Ventricle End-Diastolic Volume Index, LVESV—Left Ventricle End-Systolic Volume, LVESVI—Left Ventricle End-Systolic Volume Index, LVEF—Left Ventricle Ejection Fraction, LVM—Left Ventricle Mass, LVSV—Left Ventricle Stroke Volume, LVSVI—Left Ventricle Stroke Volume Index, RVCI—Right Ventricle Cardiac Index, RVEDV—Right Ventricle End-Diastolic Volume, RVEDVI—Right Ventricle End-Diastolic Volume Index, RVESV—Right Ventricle End-Systolic Volume., RVESVI- Right Ventricle End-Systolic Volume Index.

## Data Availability

The data presented in this study are available on request from the corresponding author. The data are not publicly available due to privacy and ethical issues.

## References

[1] Vu Q.M., Fitzpatrick A.L., Cope J.R., Bertolli J., Sotoodehnia N., West T.E., Gentile N., Unger E.R. (2024). Estimates of Incidence and Predictors of Fatiguing Illness after SARS-CoV-2 Infection. Emerg. Infect. Dis..

[2] Greenhalgh T., Manoj S., Perlowski A., Nikolich J.Z. (2024). Long COVID: A clinical update. Lancet.

[3] National Institute for Health and Care Excellence COVID-19 Rapid Guideline: Managing the Long-Term Effects of COVID-19. NICE Guideline [NG188]. https://www.nice.org.uk/guidance/ng188.

[4] CDC (2022). Post-COVID Conditions: Information for Healthcare Providers. https://www.cdc.gov/covid/hcp/clinical-overview/?CDC_AAref_Val=https://www.cdc.gov/coronavirus/2019-ncov/hcp/clinical-care/post-covid-conditions.html.

[5] Nabavi N. (2020). Long covid: How to define it and how to manage it. BMJ.

[6] Raman B., Bluemke D.A., Lüscher T.F., Neubaueret S. (2022). Long COVID: Post-acute sequelae of COVID-19 with a cardiovascular focus. Eur. Heart J..

[7] Peluso M.J., Deeks S.G. (2024). Mechanisms of long COVID and the path toward therapeutics. Cell.

[8] Klein J., Wood J., Jaycox J.R., Dhodapkar R.M., Lu P., Gehlhausen J.R., Tabachnikova A., Greene K., Tabacof L., Malik A.A. (2023). Distinguishing features of long COVID identified through immune profiling. Nature.

[9] Basso C., Leone O., Rizzo S., De Gaspari M., van der Wal A.C., Aubry M.C., Bois M.C., Lin P.T., Maleszewski J.J. (2020). Pathological features of COVID-19-associated myocardial injury: A multicentre cardiovascular pathology study. Eur. Heart J..

[10] Halushka M.K., Vander Heide R.S. (2021). Myocarditis is rare in COVID-19 autopsies: Cardiovascular findings across 277 postmortem examinations. Cardiovasc. Pathol..

[11] Kawakami R., Sakamoto A., Kawai K., Gianatti A., Pellegrini D., Nasr A., Kutys B., Guo L., Cornelissen A., Mori M. (2021). Pathological evidence for SARS-CoV-2 as a cause of myocarditis: JACC review topic of the week. J. Am. Coll. Cardiol..

[12] Canale M.P., Menghini R., Martelli E., Federici M. (2021). COVID-19–Associated Endothelial Dysfunction and Microvascular Injury. Card. Electrophysiol. Clin..

[13] Heidecker B., Libby P., Vassiliou V.S., Roubille F., Vardeny O., Hassager C., Gatzoulis M.A., Mamas M.A., Cooper L.T., Shoenrath F. (2025). Vaccination as a new form of cardiovascular prevention: A European Society of Cardiology clinical consensus statement. Eur. Heart J..

[14] Mahajan S., Kunal S., Shah B., Garg S., Palleda G.M., Bansal A., Batra V., Yusuf J., Mukhopadhyay S., Kumar S. (2021). Left ventricular global longitudinal strain in COVID-19 recovered patients. Echocardiography.

[15] Kim J., Volodarskiy A., Sultana R., Pollie M.P., Yum B., Nambiar L., Tafreshi R., Mitlak H.W., RoyChoudhury A., Horn E.M. (2020). Prognostic utility of right ventricular remodeling over conventional risk stratification in patients with COVID-19. J. Am. Coll. Cardiol..

[16] Moody W.E., Liu B., Mahmoud-Elsayed H.M., Senior J., Lalla S.S., Khan-Kheil A.M., Brown S., Saif A., Moss A., Bradlow W.M. (2021). Persisting adverse ventricular remodeling in COVID-19 survivors: A longitudinal echocardiographic study. J. Am. Soc. Echocardiogr..

[17] Schellenberg J., Matits L., Bizjak D.A., Deibert P., Friedmann-Bette B., Göpel S., Merle U., Niess A., Frey N., Morath O. (2025). Cardiac structure and function 1.5 years after COVID-19: Results from the EPILOC study. Infection.

[18] Asanin M., Ercegovac M., Krljanac G., Djukic T., Coric V., Jerotic D., Pljesa-Ercegovac M., Matic M., Milosevic I., Viduljevic M. (2023). Antioxidant Genetic Variants Modify Echocardiography Indices in Long COVID. Int. J. Mol. Sci..

[19] Lang R.M., Badano L.P., Mor-Avi V., Afilalo J., Armstrong A., Ernande L., Flachskampf F.A., Foster E., Goldstein S.A., Kuznetsova T. (2015). Recommendations for Cardiac Chamber Quantification by Echocardiography in Adults: An Update from the American Society of Echocardiography and the European Association of Cardiovascular Imaging. Eur. Heart J. Cardiovasc. Imaging.

[20] Cerqueira M.D., Weissman N.J., Dilsizian V., Jacobs A.K., Kaul S., Laskey W.K., Pennell D.J., Rumberger J.A., Ryan T., Verani M.S. (2002). Standardized myocardial segmentation and nomenclature for tomographic imaging of the heart. A statement for healthcare professionals from the Cardiac Imaging Committee of the Council on Clinical Cardiology of the American Heart Association. Circulation.

[21] Nagueh S.F., Smiseth O.A., Appleton C.P., Byrd B.F., Dokainish H., Edvardsen T., Flachskampf F.A., Gillebert T.C., Klein A.L., Lancellotti P. (2016). Recommendations for the Evaluation of Left Ventricular Diastolic Function by Echocardiography: An Update from the American Society of Echocardiography and the European Association of Cardiovascular Imaging. J. Am. Soc. Echocardiogr..

[22] Smiseth O.A., Morris D.A., Cardim N., Cikes M., Delgado V., Donal E., Flachskampf F.A., Galderisi M., Gerber B.L., Gimelli A. (2022). Multimodality imaging in patients with heart failure and preserved ejection fraction: An expert consensus document of the European Association of Cardiovascular Imaging. Eur. Heart J. Cardiovasc. Imaging.

[23] Voigt J.U., Mălăescu G.G., Haugaa K., Badano L. (2020). How to do LA strain. Eur. Heart J. Cardiovasc. Imaging.

[24] Messroghli D.R., Moon J.C., Ferreira V.M., Grosse-Wortmann L., He T., Kellman P., Mascherbauer J., Nezafat R., Salerno M., Schelbert E.B. (2017). Clinical recommendations for cardiovascular magnetic resonance mapping of T1, T2, T2* and extracellular volume: A consensus statement by the Society for Cardiovascular Magnetic Resonance (SCMR) endorsed by the European Association for Cardiovascular Imaging (EACVI). J. Cardiovasc. Magn. Reson..

[25] Shrestha A.B., Mehta A., Pokharel P., Mishra A., Adhikari L., Shrestha S., Yadav R.S., Khanal S., Sah R., Nowrouzi-Kia B. (2023). Long COVID Syndrome and Cardiovascular Manifestations: A Systematic Review and Meta-Analysis. Diagnostics.

[26] Pérez-González A., Araújo-Ameijeiras A., Fernández-Villar A., Crespo M., Poveda E. (2022). Cohort COVID-19 of the Galicia Sur Health Research Institute. Long COVID in hospitalized and non-hospitalized patients in a large cohort in Northwest Spain, a prospective cohort study. Sci. Rep..

[27] Sudre C.H., Murray B., Varsavsky T., Graham M.S., Penfold R.S., Bowyer R.C., Pujol J.C., Klaser K., Antonelli M., Canas L.S. (2021). Attributes and predictors of long COVID. Nat. Med..

[28] Huang C., Huang L., Wang Y., Li X., Ren L., Gu X., Kang L., Guo L., Liu M., Zhou X. (2021). 6-month consequences of COVID-19 in patients discharged from hospital: A cohort study. Lancet.

[29] Subramanian A., Nirantharakumar K., Hughes S., Myles P., Williams T., Gokhale K.M., Taverner T., Chandan J.S., Brown K., Simms-Williams N. (2022). Symptoms and risk factors for long COVID in non-hospitalized adults. Nat. Med..

[30] Matsumoto C., Shibata S., Kishi T., Morimoto S., Mogi M., Yamamoto K., Kobayashi K., Tanaka M., Asayama K., Yamamoto E. (2022). Long COVID and hypertension-related disorders: A report from the Japanese Society of Hypertension Project Team on COVID-19. Hypertens. Res..

[31] Lechuga G.C., Morel C.M., De-Simone S.G. (2023). Hematological alterations associated with long COVID-19. Front. Physiol..

[32] Bazdar S., Bloemsma L.D., Baalbaki N., Blankestijn J.M., Cornelissen M.E., Beijers R.J., Sondermeijer B.M., Wijck Y.V., Downward G.S., Maitland-van der Zee A.H. (2024). Hemoglobin and Its Relationship with Fatigue in Long-COVID Patients Three to Six Months after SARS-CoV-2 Infection. Biomedicines.

[33] Pasini E., Corsetti G., Romano C., Scarabelli T.M., Chen-Scarabelli C., Saravolatz L., Dioguardi F.S. (2021). Serum metabolic Profile in Patients with Long-Covid (PASC) Syndrome: Clinical implications. Front. Med..

[34] Zeng F., Huang Y., Guo Y., Yin M., Chen X., Xiao L., Deng G. (2020). Association of inflammatory markers with the severity of COVID-19: A meta-analysis. Int. J. Infect. Dis..

[35] Pober J.S., Sessa W.C. (2007). Evolving functions of endothelial cells in inflammation. Nat. Rev. Immunol..

[36] Kotecha T., Knight D.S., Razvi Y., Kumar K., Vimalesvaran K., Thornton G., Patel R., Chacko L., Brown J.T., Coyle C. (2021). Patterns of myocardial injury in recovered troponin-positive COVID-19 patients assessed by cardiovascular magnetic resonance. Eur. Heart J..

[37] Bohmwald K., Diethelm-Varela B., Rodríguez-Guilarte L., Rivera T., Riedel C.A., González P.A., Kalergis A.M. (2024). Pathophysiological, immunological, and inflammatory features of long COVID-19. Front. Immunol..

[38] Crawford A., Fassett R.G., Geraghty D.P., Kunde D.A., Ball M.J., Robertson I.K., Coombes J.S. (2012). Relationships between single nucleotide polymorphisms of antioxidant enzymes and disease. Gene.

[39] Voetsch B., Jin R.C., Bierl C., Benke K.S., Kenet G., Simioni P., Ottaviano F., Damasceno B.P., Annichino-Bizacchi J.M. (2007). Promoter Polymorphisms in the Plasma Glutathione Peroxidase (GPx-3) Gene: A Novel Risk Factor for Arterial Ischemic Stroke Among Young Adults and Children. Stroke.

[40] Jerotic D., Ranin J., Bukumiric Z., Djukic T., Coric V., Savic-Radojevic A., Asanin M., Ercegovac M., Milosevic I., Pljesa-Ercegovaca M. (2022). SOD2 rs4880 and GPX1 rs1050450 polymorphisms do not confer risk of COVID-19, but influence inflammation or coagulation parameters in Serbian cohort. Redox Rep..

[41] Puntmann V.O., Martin S., Shchendrygina A., Hoffmann J., Ka M.M., Giokoglu E., Vanchin B., Holm N., Karyou A., Laux G.S. (2022). Long-term cardiac pathology in individuals with mild initial COVID-19 illness. Nat. Med..

[42] Huang L., Zhao P., Tang D., Zhu T., Han R., Zhan C., Liu W., Zeng H., Tao Q., Xia L. (2020). Cardiac involvement in patients recovered from COVID-2019 identified using magnetic resonance imaging. JACC Cardiovasc. Imaging.

[43] Puntmann V.O., Carerj M.L., Wieters I., Fahim M., Arendt C., Hoffmann J., Shchendrygina A., Escher F., Vasa-Nicotera M., Zeiher A.M. (2020). Outcomes of cardiovascular magnetic resonance imaging in patients recently recovered from coronavirus disease 2019 (COVID-19). JAMA Cardiol..

[44] Rajpal S., Tong M.S., Borchers J., Zareba K.M., Obarski T.P., Simonetti O.P., Daniels C.J. (2021). Cardiovascular magnetic resonance findings in competitive athletes recovering from COVID-19 infection. JAMA Cardiol..

[45] Giustino G., Croft L.B., Stefanini G.G., Bragato R., Silbiger J.J., Vicenzi M., Danilov T., Kukar N., Shaban N., Kini A. (2020). Characterization of Myocardial Injury in Patients With COVID-19. J. Am. Coll. Cardiol..

[46] Li R., Wang H., Ma F., Cui G.L., Peng L.Y., Li C.Z., Zeng H.-S., Marian A.J., Wang D.-W. (2021). Widespread myocardial dysfunction in COVID-19 patients detected by myocardial strain imaging using 2-D speckle-tracking echocardiography. Acta Pharmacol. Sin..

[47] Friedrich M.G., Cooper L.T. (2021). What we (don’t) know about myocardial injury after COVID-19. Eur. Heart J..

[48] Freaney P.M., Shah S.J., Khan S.S. (2020). COVID-19 and Heart Failure With Preserved Ejection Fraction. JAMA.

[49] Smiseth O.A., Fernandes J.F., Lamata P. (2023). The challenge of understanding heart failure with supernormal left ventricular ejection fraction: Time for building the patient’s ‘digital twin’. Eur. Heart J. Cardiovasc. Imaging.

[50] Morris D.A., Ma X.-X., Belyavskiy E., Kumar R.a., Kropf M., Kraft R., Frydas A., Osmanglou E., Marquez E., Donal E. (2017). Left ventricular longitudinal systolic function analysed by 2D speckle-tracking echocardiography in heart failure with preserved ejection fraction: A meta-analysis. Open Heart.

[51] Supeł K., Wieczorkiewicz P., Przybylak K., Zielinska M. (2023). 2D Strain Analysis in Myocarditis—Can We Be Any Closer to Diagnose the Acute Phase of the Disease?. J. Clin. Med..

[52] Baycan O.F., Barman H.A., Atici A., Tatlisu A., Bolen F., Ergen P., Icten S., Gungor B., Caliskan M. (2021). Evaluation of biventricular function in patients with COVID-19 using speckle tracking echocardiography. Int. J. Cardiovasc. Imaging.

[53] Croft L.B., Krishnamoorthy P., Ro R., Anastasius M., Zhao W., Buckley S., Argulian E., Sharma S.K., Kini A., Lerakis S. (2021). Abnormal left ventricular global longitudinal strain by speckle tracking echocardiography in COVID-19 patients. Future Cardiol..

[54] Cameli M., Mondillo S., Galderisi M., Mandoli G.E., Ballo P., Nistri S., Capo V., D’Ascenzi F., D’Andrea A., Esposito R. (2017). Speckle tracking echocardiography: A practical guide. G. Ital. Di Cardiol..

[55] Bhatia H.S., Bui Q.M., King K., DeMaria A., Daniels L.B. (2021). Subclinical left ventricular dysfunction in COVID-19. IJC Heart Vasc..

[56] Tsampasian V., Bäck M., Bernardi M., Cavarretta E., Dębski M., Gati S., Hansen D., Kränkel N., Koskinas K.C., Niebauer J. (2024). Cardiovascular disease as part of Long COVID: A systematic review. Eur. J. Prev. Cardiol..

